# Expression Levels of *PPARγ* and *CYP-19* in Polycystic Ovarian Syndrome Primary Granulosa Cells: Influence of ω-3 Fatty Acid 

**DOI:** 10.22074/ijfs.2015.4240

**Published:** 2015-07-27

**Authors:** Mina Zaree, Vahideh Shahnazi, Shabnam Fayezi, Maryam Darabi, Mahzad Mehrzad-Sadaghiani, Masoud Darabi, Sajjad Khani, Mohammad Nouri

**Affiliations:** 1 Department of Biochemistry and Clinical Laboratories, School of Medicine, Tabriz University of Medical Sciences, Tabriz, Iran; 2 Women’s Reproductive Health Research Center, Alzahra Hospital, Tabriz University of Medical Sciences, Tabriz, Iran; 3 Students Research Committee, Infertility and Reproductive Health Research Center, Shahid Beheshti University of Medical Sciences, Tehran, Iran; 4 Research Center for Pharmaceutical Nanotechnology, Tabriz University of Medical Sciences, Tabriz, Iran

**Keywords:** Eicosapentaenoic Acid, PPAR Gamma, Aromatase, Granulosa Cells, Polycystic Ovary Syndrome

## Abstract

**Background:**

The omega-3 fatty acid (ω-3 fatty acid) such as eicosapentaenoic acid (EPA) is currently used in the clinic as a nutritional supplement in the treatment of poly- cystic ovarian syndrome (PCOS). The present study was designed to investigate the ef- fect of EPA on the expression levels of peroxisome proliferator-activated receptor gamma (*PPARγ*) and cytochrome P450 aromatase (encoded by the *CYP-19*) in primary cultured granulosa cells (GC) from patients undergoing *in vitro* fertilization (IVF), and also to compare these effects with those in GC of PCOS patients.

**Materials and Methods:**

In this experimental study, human GC were isolated, pri- mary cultured *in vitro*, exposed to a range of concentrations of the EPA and in- vestigated with respect to gene expression levels of *PPARγ* and *CYP-19* using real time-polymerase chain reaction (PCR). The participants (n=30) were the patients admitted to the IVF Center in February-March 2013 at Alzahra Hospital, Tabriz, Iran, who were divided into two groups as PCOS (n=15) and non-PCOS (n=15) women (controls).

**Results:**

All doses of the EPA significantly induced *PPARγ* mRNA gene expression level as compared to the control recombinant follicle stimulating hormone (rFSH) alone condi- tion. High doses of EPA in the presence of rFSH produced a stimulatory effect on expres- sion level of *PPARγ* (2.15-fold, P=0.001) and a suppressive effect (0.56-fold, P=0.01) on the expression level of *CYP-19*, only in the PCOS GC.

**Conclusion:**

EPA and FSH signaling pathway affect differentially on the gene ex- pression levels of *PPARγ* and *CYP-19* in PCOS GC. Altered FSH-induced *PPARγ* activity in PCOS GC may modulate the *CYP-19* gene expression in response to EPA, and possibly modulates the subsequent steroidogenesis of these cells.

## Introduction

Polycystic ovarian syndrome (PCOS) is the most commonly occurring cause of female infertility (1). In PCOS, there is an imbalance of female sex hormones, which may lead to ovarian cysts and irregular or absent menstrual cycle. The abnormality has been mainly attributed to the suppression of the follicle stimulating hormone (FSH) secretion by an excess androgen produced from the ovary. Accelerated early follicular growth leads to attenuated FSH responsiveness and the premature luteinisation of granulosa cells (GC). In turn, the development of the dominant follicle is disrupted which causes cystic follicular arrest (2).

The cytochrome P450 aromatase, encoded by the *CYP-19* gene, in ovarian GC that converts testosterone to estradiol is induced by FSH during early follicle development. The timely expression of *CYP-19* in GC plays a critical role in follicle development. In the *CYP-19* knockout mice, antrum formation is arrested at a stage before ovulation and no corpora lutea are formed (3). The follicular arrest of PCOS has been characterized by the lack of *in vivo* FSH-induced *CYP-19* activity in GC (4).

The expressions of *CYP-19* is coordinately regulated and efficiently inhibited by thiazolidinediones (TZDs) in human GC obtained from *in vitro* fertilization (IVF) (5, 6). TZDs are known as agonists of the gamma isoform of the peroxisome proliferatoractivated receptor (*PPARγ*), a family of nuclear receptors regulating the expression of genes involved in lipid metabolism, insulin sensitivity, and cellular differentiation. *PPARγ* expression has been found in the GC (7). The *PPARγ* may regulate the steroidogenesis, thereby contributes to the regulation of ovarian function (8). Previous studies have reported that retinoid X receptor (RXR) response elements are present in the *CYP-19*; however, no exact region that responds independently to *PPARγ* has yet been identified (9).

There is a strong indication that omega-3 fatty acids (ω-3 fatty acids) have protective action against PCOS (10). In particular, eicosapentaenoic acid (EPA), a long-chain ω-3 fatty acid (PUFA), is a natural high-affinity ligand for *PPARγ*. Despite the increasing clinical use, the mechanisms by which EPA exerts its effects is yet relatively unknown. The aim of the present study was to investigate the effects of EPA on gene expression levels of *PPARγ* and *CYP-19* in cultured GC from patients undergoing IVF, and also to compare these effects with those in GC of PCOS patients.

## Materials and Methods

This experimental study was approved by the Ethics Committee of Tabriz University of Medical Sciences. All patients gave a written informed consent and their confidentiality and anonymity were protected.

### Primary cell culture

Sampling was done by a simple consecutive method covering all patients (n=30) who were admitted to the IVF Center in February-March 2013 at Alzahra Hospital, Tabriz, East Azerbaijan Province, Iran. PCOS were defined as the presence of 12 or more follicles measuring 2-9 mm with clinical (a Ferriman–Gallwey score >7) and/or biochemical hyperandrogenism (total testosterone >3 nmol/l) (11). The participants (n=30) were divided into two groups as PCOS (n=15) and non-PCOS (n=15) women (controls).

Inclusion criteria were no alcohol consumption and no smoking habit. Uterus abnormalities, endometriosis, anovulation, positive history of endocrine disease and inflammatory disorders such as thyroid and adrenal disorders, hormonal treatment, and history of recurrent infections were considered as exclusion criteria in this study. Control group (n=15) included individuals with age- (27.62 ± 4.14 years) and body mass index (BMI)- (25.11 ± 2.57 kg/m_2_) matched with no evidence of hyperandrogenemia or menstrual irregularities. All patients underwent a standard infertility evaluation, including hormonal testing and assessment of the uterus and fallopian tubes by means of hysterosalpingography. Patients underwent a long gonadotropin-releasing hormone (GnRH) agonist (decapeptyl, Debio Pharm, Geneva, Switzerland)/ FSH-long down regulation protocol as described previously by us (12). GC was isolated from aspirated follicular fluid by hyaluronidase digestion, followed by Percoll gradient centrifugation (13).

Three sets of experiments with both PCOS and control groups were performed. GC was pooled because the number of cells from follicles was insufficient to perform individualized culture. In the experiments, each group composed of GC pooled from 5 women. In total, GC were isolated and pooled from 15 PCOS and 15 control women of reproductive age. The GC were counted with a homocytometer, and approximately 1×10^6^ cells were plated in a 12-well culture plate containing dulbecco’s modified eagle medium/ nutrient mixture/F-12 (DMEM/F12, Cellgro, USA) medium supplemented with 10% fetal bovine serum (FBS), 100 IU/ml penicillin, and 100 μg/ml streptomycin, for 24 hours. Cells were maintained at 37˚C in 5% CO_2_ in a humidified incubator. EPA (Sigma, St. Louis, MO) was conjugated with bovine serum albumin (BSA) fatty acid-free (Sigma, St. Louis, MO) before treatment (14). GC, after serum starvation overnight, were treated with indicated concentrations of EPA (25-100 μM), both either with or without pretreatment with recombinant (r)FSH (100 ng/mL).

### Real-time polymerase chain reaction analysis

Total RNA was isolated using RNX-Plus according to the instructions of the manufacturer. RNA pellets were ethanol-precipitated, washed, and resuspended in sterile ribonuclease-free water. Two μg of total RNA were reverse transcribed into cDNA using SuperScript II reverse transcriptase (Life Technologies, Carlsbad, CA, USA). Real-time polymerase chain reaction (PCR) was carried out using the fluorescent dye SYBR-Green and a Bio-Rad CFX real-time PCR system (BioRad Co, CA, USA). The primers used for qPCR were as follows: *PPARγ*, 5΄ ATGACAGACCTCAGACAGATTG 3΄ (sense) and 5΄ AATGTTGGCAGTGGCTCACGTG 3΄ (antisense); *CYP-19*, 5΄ TCTTGGTGTGGAATTATGAG 3΄ (sense) and 5΄ TTGAGGACTTGCTGATAATG 3΄ (antisense); glyceraldehydes 3-phosphate dehydrogenase (*GAPDH*), 5 AAGCTCATTTCCTGGTATGACG 3 (sense) and 5΄ TCTTCCTCTTGTGCTCTTGCTGG 3΄ (antisense).

Samples were assayed in duplicates. The amount of specific PCR products was normalized to the *GAPDH* mRNA content, and quantities were expressed as an x-fold difference relative to a control.

### Statistical analysis

Values are presented as mean ± standard deviation (SD) of 3 separate experiments done in duplicate. Data in all groups were normally distributed. Statistically significant differences in mean values between groups were assessed by t tests. Analysis of variance test were used for comparing the group means. Calculation of significance between groups was done according to analysis of variance (ANOVA) with post hoc Tukey’s tests for multiple comparisons. Repeated- measures ANOVA was used for measures of response times, and a P value of <0.05 was considered statistically significant.

## Results

**Fig.1:**
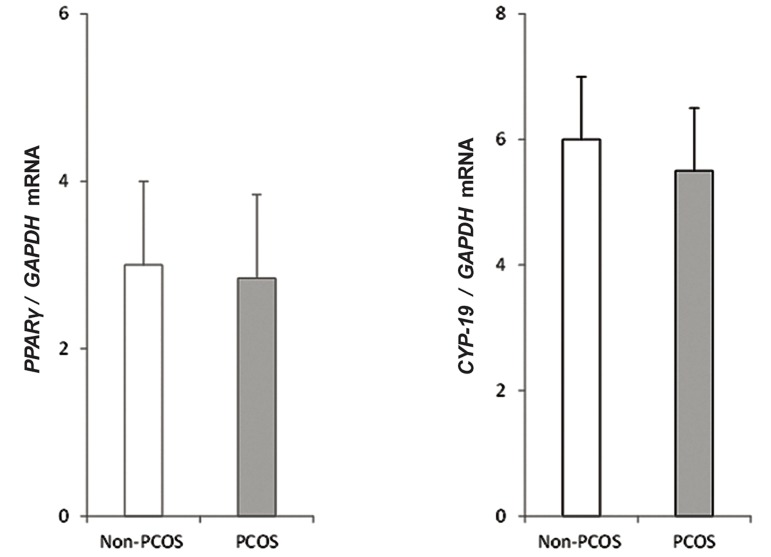
Quantitative analysis of *PPARγ* (A) and *CYP-19* (B) genes expression levels by real-time PCR in GCs from PCOS and non PCOS-women. Each expression level was normalized to the *GAPDH* levels. The mean ± SD of three independent determinations with cells pooled from 5 women per group per experiment (t test). PCR; Polymerase chain reaction, GCs; Granulosa cells and PCOS; Polycystic ovarian syndrome.

Figure 1 shows the genes expression levels measured by quantitative PCR method in GC from patients with PCOS and non-PCOS women. Primarily, no significant differences were found in the gene expression levels of *PPARγ* and *CYP-19* between the two groups.

**Fig.2:**
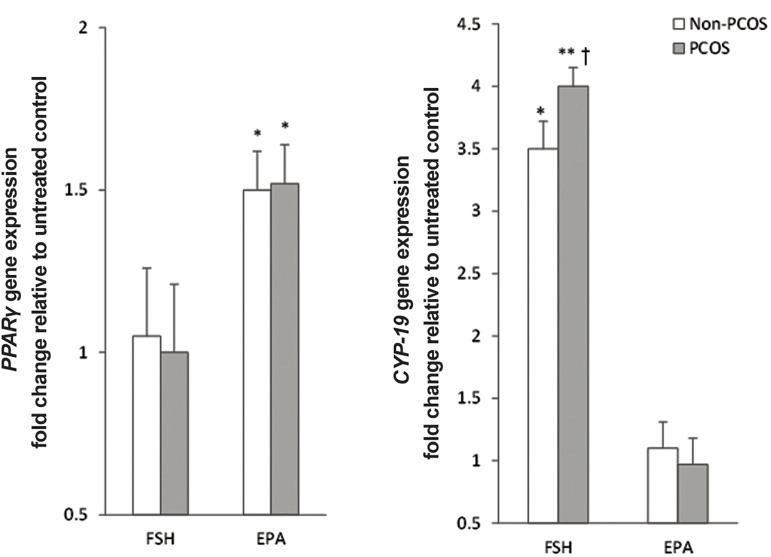
Effect of the follicle stimulating hormone (FSH) and eicosapentaenoic acid (EPA) incubation on mRNA expression levels of *PPARγ* and *CYP-19*. GCs, after serum starvation, were incubated for 24 hours ± 100 ng/mL FSH or 50 μmol/L EPA. Cell lysates were prepared and analyzed by real-time PCR for genes expression levels. Expression levels of *PPARγ* (A) and *CYP-19* (B) in each lysate were normalized to the amount of *GAPDH* and represented as fold of untreated control. The mean ± SD of three independent experiments with cells pooled from 5 women per group per experiment (t test). *; P<0.05 and **; P<0.01 vs. untreated control and †; P<0.05 vs. non-PCOS. PCR; Polymerase chain reaction, GCs; Granulosa cells and PCOS; Polycystic ovarian syndrome.

To determine the effect of rFSH stimulation on expression levels of *PPARγ* and *CYP-19*, GC was treated with rFSH. Only *CYP-19* showed a significant increase in mRNA level (P<0.001, Fig.2), which was more elevated in PCOS than in non- PCOS (mean 4.0-fold vs. 3.5-fold, respectively, P=0.03). In contrast, incubation with EPA alone resulted in comparable upregulation of *PPARγ* expression level (1.49 ± 0.12 vs. 1.52 ± 0.11, P=0.51) in GCs from non-PCOS and PCOS patients. However, no such changes were observed for *CYP-19* expression level in EPA-treated cells (Fig.2).

**Fig.3:**
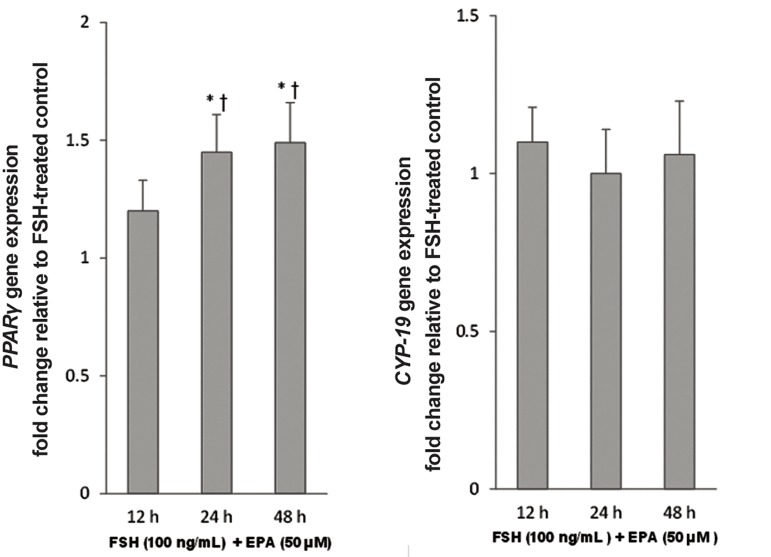
Effect of eicosapentaenoic acid (EPA) incubation time on mRNA expression levels of *PPARγ* and *CYP-19*. GCs, after serum starvation, were incubated in 100 ng/mL follicle stimulating hormone (FSH) alone or in combination with 50 μmol/L EPA for 12 hours, 24 hours and 48 hours. Cell lysates were prepared and analyzed by real-time PCR for genes expression levels. Expression levels of *PPARγ* (A) and *CYP-19* (B) in each lysate were normalized to the amount of *GAPDH* and represented as fold of FSH-treated control. The mean ± SD of three independent experiments with cells pooled from 5 women per group per experiment (repeated-measures ANOVA. *; P<0.05 and †; P<0.05 vs. FSH-treated control and 12-hour incubation, respectively). PCR; Polymerase chain reaction, GCs; Granulosa cells and h; Hours.

Comparison of control rFSH with the combined rFSH-EPA condition showed a similar response compared to the EPA alone. To optimize the assay, cultured GC from non PCOS women were incubated with the 50 μmol/L EPA and the incubation time ranged from 12 hours to 48 hours. While no significant changes were observed in the expression level of *CYP-19*, the expression level of *PPARγ* increased by 30% (P=0.02) after 24 hours. However, later no further changes were observed in the expression levels of both mRNAs (Fig.3).

**Fig.4:**
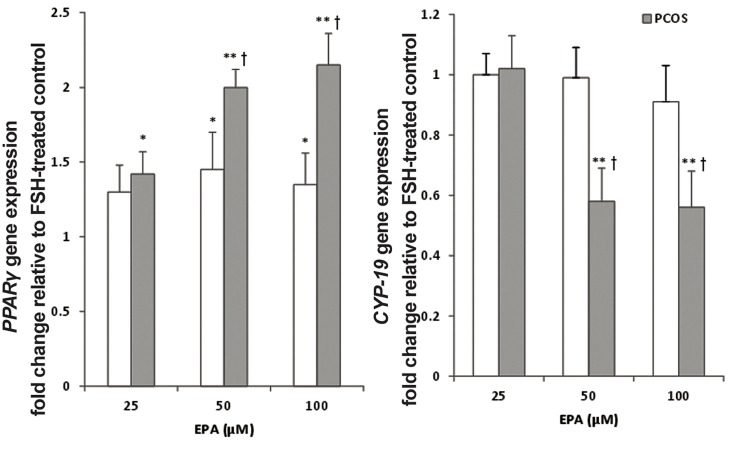
Effect of different doses of eicosapentaenoic acid (EPA) on expression levels of *PPARγ* and *CYP-19* in follicle stimulating hormone (FSH)-stimulated GCs from PCOS and non-PCOS women. GCs, after serum starvation, were incubated in 100 ng/mL FSH alone or in combination with 25 μmol/L, 50 μmol/L or 100 μmol/L EPA for 24 hours. Cell lysates were prepared and analyzed by real-time PCR for genes expression levels. Expression levels of *PPARγ* (A) and *CYP-19* (B) in each lysate were normalized to the amount of *GAPDH* and represented as fold of FSH-treated control. The mean ± SD of three independent experiments with cells pooled from 5 women per group per experiment (ANOVA with post hoc Tukey’s test, *; P<0.05, **; P<0.01 vs. FSH-treated control and †; P<0.01 vs. non-PCOS). PCR; Polymerase chain reaction, GCs; Granulosa cells and PCOS; Polycystic ovarian syndrome.

In the next series of experiments, three doses of EPA (0-100 μM) were tested in the presence of rFSH. Treatment of GCs with 50 and 100 μM doses of the EPA significantly increased *PPARγ* mRNA gene expression level compared to the control rFSH alone condition (P<0.05). *PPARγ* displayed a larger fold change in the PCOS group than in the non-PCOS group. The magnitude of this difference between non-PCOS and PCOS was more pronounced at the higher doses of EPA (e.g., 1.42- fold at 25 μmol vs. 2.15-fold at 100 μM, P=0.008). Moreover, it was identified that the expression level of *CYP-19* was also influenced by the higher doses of EPA in the PCOS GC as compared to the control. The combination of high doses of EPA in the presence of rFSH produced a strong suppressive effect on the *CYP-19* gene expression level in the PCOS GC (0.56-fold, P=0.01, Fig.4).

## Discussion

PPAR-γ has been shown to be critically important in multiple biological functions such as fertility (12), while EPA and docosahexanoic acid (DHA) are natural, preferentially-binding ligands for this receptor. It has been shown that EPA and DHA down-regulate activation of NF-κB through increasing both PPAR-γ mRNA levels and protein activity in different types of cells. These effects may be one of the underlying mechanisms for the anti-inflammatory effect of the ω-3 PUFA (15, 16). To the contrary, although no change in *PPARγ* mRNA expression level has been reported previously in certain types of cells after exposure to EPA (17). Our results demonstrated that there were mRNA expression levels of *PPARγ* and *CYP-19* in pre-ovulatory human GC, and that *PPARγ* was increased by EPA. This suggests that EPA may elicit important biological responses in GC via activation of *PPARγ*.

*PPARγ* is a key transcription factor involved in follicular differentiation (18) and ovarian GC tumor (19). It has been shown that a decrease in expression level of *PPARγ* in response to luteinizing hormone (LH) is important for ovulation and/ or luteinization. GC differentiation into the corpus luteum in response to the LH surge is accompanied by reduced *CYP-19* activity. It has been reported that the expression level of mRNA for *PPARγ* in follicles is inversely related to the expression level of mRNA for *CYP-19* (20). Overexpression of *PPARγ* in the KGN ovarian granulosa-like tumor cell line reduced FSH-stimulated *CYP-19* mRNAs (21). These observations suggest that *PPARγ* has an inhibitory effect on the *CYP-19* activity as well as on ovulation and/or luteinization. The complete disruption of FSH-induced estradiol production by synthetic PPAR-γ agonists in cultured human ovarian cells has been attributed to *CYP-19* (5). It has been shown that *PPARγ* agonists suppress the *CYP-19* mRNA expression level in human GC, in a dose-dependent manner, probably via nuclear receptor system *PPARγ*: RXR heterodimer (22). However, the data reported in the literature about the effects of TZDs on *CYP-19* activity in the ovary are controversial. Either no effect (23) or suppressive effects (22) have been shown, which could partly be attributed to a variety of *PPARγ* independent signaling events (24). Furthermore, no specific data is available regarding the effect of either the synthetic or natural *PPARγ* agonists on the expression and activity of GC aromatase in PCOS.

As shown herein and reported previously, FSH induces the expression level of *CYP-19* (25, 26). In contrast, levels of mRNA for *PPARγ* were not affected by treatment with rFSH, in agreement with the observations made previously in rats (27). Cotreatment with EPA and rFSH resulted in enhanced *PPARγ* expression level both in control and PCOS GC. However, altered levels of gene expression in PCOS granulosa in response to the combined drug condition was not similar to that observed in control granulosa. In cultured GC obtained from patients with PCOS, EPA induced a more pronounced effect with rFSH treatment on the mRNA expression level of *PPARγ*. Furthermore, EPA treatment of PCOS GC remarkably down regulated *CYP-19* gene, as compared with non-PCOS patients. Coffler et al. have shown that women with PCOS exhibited dose-dependent GC hyperresponsiveness to FSH and increased production of estradiol (28, 29). The above results implied a possibility that the apparent suppressive effect of EPA on hypersensitivity of PCOS GC to rFSH may be due to a negative regulation of the rFSH signaling by activated *PPARγ*. Accordingly, *CYP-19* down-regulation via induction of *PPARγ* has recently been noted in GC from subjects undergoing IVF (21).

The deregulated synthesis of estradiol (E_2_) by PCOS GC has been associated with the arrest of early antral follicle development (30). The GC from PCO antral follicles produce normal or increased E_2_ amounts *in vitro* (31), even though follicles in women with PCOS contain low levels of *CYP-19* mRNA (32). This would suggest an in vivo blockade of estrogen production by follicular environment in PCOS. This is in accordance with our findings of no statistically significant difference in the expression of *CYP-19* in primary culture between GC from patients with PCOS and those from control non-PCOS.

Unlike the response to combination of rFSH and EPA, the gene expression of *PPARγ* in response to EPA alone was not different between control and PCOS GC. On the other hand, rFSH alone exerted no apparent effect on *PPARγ* gene expression level in the both control and PCOS GC. Based on these results, the higher EPA-induced *PPARγ* expression level in PCOS than in control GC may be somewhat explained by concomitant hypersensitivity of PCOS cells to FSH. FSH activates several signaling mechanisms through its surface G proteincoupled receptor (GPCR) such as the MEK and PI3K pathways, which are potentially involved in the regulation of *PPARγ*-mediated signaling in GCs (33).

Several clinical evidences support the preventive and therapeutic effects of ω-3 fatty acids in menopausal problems (10). Recently, ω-3 fatty acids supplementation has been related to the improvement in insulin sensitivity (34), and less androgenic and atherogenic lipid profiles (35) in women with PCOS. The results of the present study confirmed the potential effect of ω-3 fatty acids on the ovulatory function of PCOS. It is suggested that the modulatory effect of ω-3 fatty acids on the GC steroidogenesis could also play an important role in the oocyte maturation and subsequent ovulation.

Although previous research has shown beneficial effect of *PPARγ* agonists in PCOS, this is the first study to examine the combined effect of EPA and rFSH on the gene expression levels of *PPARγ* and *CYP-19* in human GC. The small sample size, pooled estimate and lack of assessment of *CYP-19* activity may be seen as limitations of this study. However, the regulatory effects were simultaneously analyzed by studying the expression level in control and PCOS GC, which made it possible to identify similarities and differences. Since the preliminary findings of the present study were derived from cultured GC, it remained to confirm the in vivo effect of EPA and to further assess the possible mechanism of action of EPA in the treatment of PCOS.

## Conclusion

Our study showed that EPA and FSH signaling pathway affect differentially on the gene expression levels of *PPARγ* and *CYP-19* in PCOS GC. We speculated that altered FSH-induced *PPARγ* activity in PCOS GC may modulate the *CYP-19* gene expression level in response to EPA, and subsequently modulates the steroidogenesis of these cells.
